# The impact of digital empowerment on open innovation performance of enterprises from the perspective of SOR

**DOI:** 10.3389/fpsyg.2023.1109149

**Published:** 2023-02-08

**Authors:** Liang Lingling, Li Ye

**Affiliations:** School of Economics and Management, Shanghai Institute of Technology, Shanghai, China

**Keywords:** digital empowerment, open innovation, cognitivism, SOR theory, NCA

## Abstract

**Introduction:**

As China’s digital transformation index continues to climb and market openness increases, the active implementation of open innovation embedded in digital innovation eco-networks is key to implementing sustainable innovation-driven strategies. The widespread use of digital technologies has broken through the traditional closed boundaries of enterprises and enhanced technology exchange, information communication and R&D collaboration with other innovation agents.However, many enterprises’ digital empowerment efforts only stay at the level of digital technology, but do not rise to the level of corporate strategy. How to comprehensively promote the change of enterprise digital empowerment and help enterprises build a sustainable open innovation ecosystem needs further research.

**Methods:**

This article uses the structure equation model and the necessary condition analysis methods to combine the stimulus-organization-reaction (SOR) theory to analyze the conduction mechanism of digital authorization to open innovation from a cognitive perspective.

**Results:**

(1) In the era of digital economy, digital empowerment emphasizes the initiative and adaptability of enterprises, and explores a sustainable digital road suitable for enterprises themselves; (2) Organizational emotional ability and organizational disordered atmosphere play a mediating role between digital empowerment and open innovation, but organizational emotional ability has a positive impact on open innovation, while organizational disordered atmosphere is the opposite. (3) Organizational identity positively regulates the relationship between the disordered atmosphere and open innovation.

**Discussion:**

The development of digital technology has adapted deviations with traditional management models. Organizing the investment in digital construction should also pay attention to the digital training and digital thinking of organizational members.Organizations should provide organizational support through various channels, enhance employees’ organizational commitments to create a relationship -shaped psychological contract, regularly carry out digital education and organizational culture, reduce the differential atmosphere between teams, enhance the team’s awareness of cooperation and trust in the teamAnd overall consciousness.

## Introduction

China’s 14th Five-Year Plan outlines that it will give full play to the advantages of resources and scenarios, promote the deep integration of the digital economy and the real, and empower traditional industries to transform and upgrade. The 2021 Accenture China Enterprise Digital Transformation Index Research Report states that the average score of China’s Enterprise Digital Transformation Index rose from 37 in 2018 to 54, and the digitalization level of China’s enterprises continues to climb. In the context of Industry 4.0 era, the new generation of digital technologies represented by big data, artificial intelligence and deep learning deeply integrate the enterprise management and innovation process, gradually overturning the traditional enterprise management process and innovation model (Yoo et al., 2012). The strong linkage and convergence of digital technologies have overturned the traditional industrial form, breaking through the traditional industrial cluster model with high dependence on geographical proximity and gradually evolving toward virtual network clustering, blurring industrial boundaries; self-growth has reconfigured the enterprise innovation process, shortening the innovation cycle and increasing the speed of iteration; reprogrammability has accelerated the flow of information and knowledge, enhancing the self-renewal of enterprises and the ability to exchange resources with stakeholders instantly. Instantaneous exchange of resources with stakeholders (Nambisan et al., 2017). However, in the face of the digitalization wave, many SMEs’ digitalization process only stays at the stage of buying equipment and robots, and managers’ understanding of digital empowerment is very superficial and mechanical, most of them are in a “half-baked” state, and the digital empowerment process is daunting and on the sidelines (Wimelius et al., 2021). Most companies are in a “half-assed” state, and the digital empowerment process is shy and on hold. According to a McKinsey report, the probability of failure of digital transformation in general reaches 80%, and the deeper enterprises enter the digital transformation area, the more they will realize the difference and disconnect between digital transformation theory and reality (Smith and Beretta, 2021). Obviously, in the process of digital empowerment, as enterprises digitize more and more deeply, how can enterprises digitally empower themselves to smoothly pass through the deep water and achieve the perfect crossing of the valley of death, so as to enhance open innovation performance? In the face of high digital transformation failure rate, how can enterprises as the main body of transformation play an active role in the organization, how to achieve “internalization of digital empowerment” from “fetishism,” from “art” and “way” to achieve adaptive innovation performance? How to realize the adaptive transformation from the “art” to the “way”? These are the two theoretical questions that this paper wants to answer.

First, the existing literature mostly focuses on the digital technology and enterprise capability aspects of digital empowerment, but less on the initiative, dynamism and self-adaptability of the organizational body in the process of digital empowerment from the perspective of organizational management. The development of digital economy has driven changes in the way innovation resources are allocated, innovation subjects and innovation organization (Hund et al., 2021) Secondly, most of the existing literature focuses on the single impact (positive or negative) of digital technology, but less on the double-edged effect of digital empowerment. For example, In a comparative analysis between developed and developing countries, ICT investment contributes more to economic growth than physical and human investment, and that developing countries are using this digital dividend to narrow the “digital divide” with developed countries (Barba-Sánchez et al., 2018). Srivastava constructs the “IT capital-IT institutions-innovation productivity” model based on the cognitive path dependence model by introducing North’s new institutional economics, Hayek’s mental model and Bandura’s explanation of learning (Srivastava, 2021). The key to the strategy and importance of resources is their scarcity rather than their universality, and that the spread and widespread use of digital technology has weakened the competitive advantage of digital resources (Carlaw and Oxley, 2008; Acemoglu et al., 2014). The popularity and widespread use of digital technology has weakened the competitive advantage of digital resources (Wimelius et al., 2021). Gebauer et al. argue that digital operations have a dampening effect on firms’ real surplus management activities (Gebauer et al., 2020). Then, regarding the issue of double-edged effects of digital empowerment, the existing literature mostly focuses on the perspectives of data leakage, data ethics and user privacy, and less explores the issue of negative effects brought by digital empowerment externalities from the perspective of organizational management. Finally, the existing literature mostly explores the negative effects of digital empowerment from the perspective of resource base theory (Lioukas et al., 2016), Theories of dynamic capabilities (Irfan et al., 2019; Schulze and Brusoni, 2022), value co-creation theory (Chen et al., 2012; Kohtamäki and Rajala, 2016; Uppström and Lönn, 2017; Shin et al., 2020), organizational learning theory (Koo et al., 2017; Deperi et al., 2022) and knowledge management theory (Edwards, 2022; Saratchandra et al., 2022). The analysis of innovation performance paths in digitally empowered enterprises has been conducted from different perspectives, and the formation mechanism of open innovation paths in enterprises has been less explored from the cognitivist learning theory (SOR framework).

SOR theory is a psychological model based on cognitivism (Han et al., 2022). Unlike behaviorism, cognitivism believes that “epiphany” is the primary source of learning, rather than exploration and error (Watson and Coulter, 2008; Chen and Krajbich, 2017). Cognitivism rejects the idea that there is a relationship between stimulus and response. Cognitivism rejects the direct, mechanical, and passive nature of the connection between stimulus and response and emphasizes the subjective and constructive nature of the organism (Kamboj et al., 2018; Hsiao and Tang, 2021). Digital empowerment is a systematic process of enterprise self-transformation, using new generation digital technology as the means of empowerment, triggering all-round changes from the inside out, disrupting enterprise innovation processes, behavioral logic and ecological networks, and reshaping enterprise competitive advantage and value chain status (Li et al., 2022; Yang et al., 2023). We argue that digital empowerment is a way of survival that enterprises actively choose to cope with environmental uncertainty in the VUCA era. Based on the cognitivist learning theoretical framework (SOR theoretical framework), we focus on the “digital empowerment-open innovation” relationship through the integration and expansion of digital empowerment and open innovation. This paper explores the mechanism of organizational identity to regulate the “organism-response” process in a complex environment through the integration and expansion of digital empowerment and open innovation, focusing on the organism (O) cognitive process in the “stimulus–response” relationship. Based on primary and secondary data from companies in different industries, this paper applies structural equation modeling and cognitivist learning theory to analyze the mechanisms and pathways of digital empowerment on open innovation performance, hoping to provide useful references for corporate innovation management.

## Literature review and research hypothesis

### The effect of digital empowerment on open innovation performance

“The term “empowerment” is derived from the term “empowerment,” which means giving employees more power to better achieve organizational goals and is a process of decentralization (Motamarri et al., 2022; Kanjanakan et al., 2023). The term “empowerment” refers to the process of empowering employees to better achieve organizational goals. Digital empowerment refers to the process in which enterprises use digital technology as an empowering means and the enterprise’s basic digital facilities, organizational management, and business processes as empowering objects, triggering comprehensive changes in resource allocation, business processes, organizational structure, and management, etc., and mobilizing the enterprise’s initiative from the inside out to adapt to the rapid changes in the external dynamic environment to gain competitive advantages (Wu et al., 2022). Empowerment is a sustainable and systematic process. As the level of digital empowerment of enterprises continues to improve, employees, teams and organizations are faced with the subversion of traditional thinking and the reshaping of digital thinking (Leyer et al., 2019). Liao et al. from the perspective of complementarity theory, this paper explains the co creation mechanism of brand community value in the context of digital enabling multimedia social networking (Liao et al., 2022).

In this paper, digital empowerment refers to the process of using digital technology as the means to empower enterprises, and using enterprise infrastructure digital facilities, organizational management and business processes as the empowerment objects, triggering comprehensive changes in resource allocation, business processes, organizational structure and management, and mobilizing enterprise initiatives from the inside out to adapt to the rapid changes in the external dynamic environment to gain competitive advantages. Empowerment is a sustainable and systematic process. As the level of digital empowerment of enterprises continues to increase, employees, teams and organizations are faced with the subversion of traditional thinking and the reshaping of digital thinking, which makes enterprises face increased organizational risks. From the perspective of organizational management theory, digital technology empowerment of individuals can easily lead to the emergence of “strong individuals,” threatening organizational stability and legitimacy, and increasing employee mobility. In summary, digital empowerment has a double-edged effect (Reischauer and Ringel, 2022). Digital empowerment is a kind of innovation, and innovation means cost and risk. And the cost, risk and benefit of promoting digital empowerment are uncertain (Quinton and Simkin, 2017). This paper combines the uncertainty and risk of digital empowerment to analyze digital empowerment from the perspective of strategy, operation, and function.

1. From a strategic perspective, as digitalization continues to penetrate into the field of enterprise management, organizations are gradually evolving in the direction of borderless (Li et al., 2018), and traditional closed organizations are evolving into multi-border organizations, multi-business entities and cross-domain organizations, and organizational assemblability and patchability, employee shareability and capacity integration have overturned the traditional management model (Pananond et al., 2020). Strategy Empowerment helps companies develop digital strategies and provides them with the direction and vision for digital innovation (Hund et al., 2021).
2. Analyzed from an operational perspective, digital empowerment transforms the traditional business processes and procedures of an enterprise to achieve operational digital empowerment. Operational empowerment refers to the process of using digital technology to transform traditional business activities into digital business activities by enterprises built on a process-oriented paradigm (Roscoe et al., 2019; Friedrich and Hoel, 2021). The process of using digital technology to transform traditional business activities into digital business activities. Operational enablement helps companies to improve internal efficiency and control costs, thus facilitating their innovation activities, and this effect is more evident in start-ups and small companies than in large companies (BarNir et al., 2003). This effect is more pronounced in start-ups and small companies than in large companies. Operations enablement enhances business process management capabilities, including increased agility and sensitivity, and organizational responsiveness (Ali and Govindan, 2021).
3. Analyzed from the functional perspective, the resource-based view believes that digital empowerment is the collection and coding of enterprise basic resources to realize resource datafication empowerment (Stylos et al., 2021; Bag et al., 2023). Resource datafication refers to taking heterogeneous and non-heterogeneous resources owned by enterprises as production factors, using digital technology as an empowering means and driving force, improving the degree of data coding and digital network of resources through the deep integration of digital technology and enterprise resources, and accelerating the reconstruction of enterprise resource integration efficiency and management mode (Beverungen et al., 2022; Quach et al., 2022). Digital empowerment greatly simplifies the daily affairs of enterprises, improves the efficiency and motivation of grassroots employees, and reduces the transaction costs of enterprises (Peng and Tao, 2022).

Based on this, this paper proposes hypothesis 1: digital empowerment has a positive effect on open innovation (H1).

### Cognitivism and the SOR theoretical framework

The “stimulus (S)-organism (O)-response (R)” model is an environmental psychology model proposed by Mehrabian and Russell based on the “stimulus (S)-response (R)” model of behaviorist psychology (Guo et al., 2021). It is a model of environmental psychology based on the behaviorist psychology of stimulus (S)-response (R). Existing studies have interpreted the “S-O-R” model from the perspectives of behaviorism, cognitivism, environmental psychology, educational psychology, management psychology, and consumer behavior patterns (Liu et al., 2022; Peng and Yue, 2022; Zubair et al., 2022).

This paper draws on cognitivist learning theory, management psychology and organizational management theory to argue that in the digital economy, digital empowerment is a stimulus for organizations to choose digital empowerment at a corresponding cost on their own initiative in the face of the risks and opportunities in the era of external digital intelligence VCUA. digital empowerment, a stimulus, in the process of organizational digital empowerment, due to its double-edged effect of enhancing the efficiency of business operations while also Based on cognitivism theory, the organization’s response to stimulus or environment is not governed by convention but guided by the subject’s expectation, and the organization’s subjective initiative and anti-vulnerability are the keys for the organization to perceive the opportunities and threats of digital empowerment and avoid the pitfalls of digital transformation. That is, stimulus refers to the organization’s active choice of digital empowerment influenced by environmental turbulence; organism refers to the organization’s internal cognition and state with subjective motivation and anti-vulnerability; and reaction refers to the performance results of organizational behavior. Among them, the research on the mediating role of organism is explored in this paper mainly from organizational identity (individual and organizational interaction perspective), organizational Chaxu atmosphere (organizational team circle perspective), and organizational emotional competence (overall organizational perspective).

### Organizational Chaxu climate and open innovation

The organizational climate of Chaxu is an organizational climate in which individuals treat members of the organization differently due to differences in the perceived degree of closeness of relationships around the core of resources or information they own or hold (Vveinhardt and Bendaraviciene, 2022). The difference in organizational climate is an organizational climate in which individuals treat members of the organization differently due to differences in perceived closeness. Existing research on differential climate is mainly from the perspective of individual employees and organizations or teams. Based on social cognitive theory and social exchange theory, different employees have different perceptions and judgments of leadership style, organizational culture and leader-employee information exchange process due to their different educational experiences, upbringing and existing perceptions, and thus have different behavioral expressions of organizational disordered climate (Chan et al., 2013). Pecino et al. argue that a positive organizational climate reduces role stress and thus increases job satisfaction among public employees (Pecino et al., 2019). Based on the gender discrimination perspective, Ciftci et al. argue that when women perceive that the organizational climate is tolerant of gender discrimination, they will come together with other women (Ciftci et al., 2020). Unlike abusive management, organizational disorderly climate is rooted in traditional Chinese culture and moral values, and the expression of relatively hidden and invisible reciprocity is rooted in organizational disorderly climate. Based on the organizational perspective, Al-Kurdi et al. argue that organizational leadership and trust in the organizational climate have a positive effect on knowledge sharing (Al-Kurdi et al., 2020). Based on the above analysis, due to the high cost and scarcity of digital empowerment, organizations tend to treat the allocation of digital empowerment resources and knowledge differently, and some mainstream businesses tend to get more digital transformation opportunities and resources, resulting in resource redundancy in mainstream business resources; while some other This seriously aggravates the inequality between organizations (Ren and Chadee, 2017), and is more likely to cause the fragmentation and information asymmetry between mainstream and non-mainstream businesses, which easily forms knowledge and information dissemination barriers, thus reducing the efficiency of organizational information and knowledge dissemination and is not conducive to organizational innovation performance. Dong et al. explores the relationship between negative news reports and financial performance from the perspective of external relations (Dong et al., 2022). But the enterprise negative news often can cause the impact to the organization atmosphere.

Based on this, this paper proposes hypothesis 2 Chaxu climate plays a mediating role between digital empowerment and open innovation (H2).

### Organizational identity and open innovation

**Figure 1:**
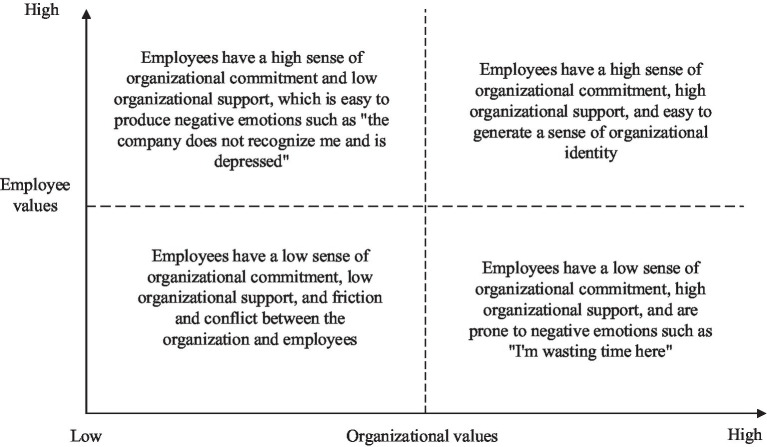
Concept of organizational identity.

Based on social identity theory, organizational identity refers to the consistency of matching the individual identity value identity of employees with the overall identity value identity of the organization (Ashforth and Mael, 1989). In addition, employees with high organizational identity tend to reach a relational psychological contract with the organization, forming a sense of responsibility and mission with the organization’s “honor and shame, and common destiny”(Lu et al., 2016). As shown in Figure 1, organizational identity has the potential to be a key factor in the development of employees (Shen, 2022). As shown in Figure 1, organizational identity has the double consistency property of organizational values and employee values. Therefore, the factors affecting organizational identity are discussed in two dimensions: individual and organizational. According to self-categorization theory and social identity theory, in the individual dimension, organizational identity has a strong subjective and emotional tendency, and individuals recognize and appreciate organizational values and organizational culture, and due to individual differences and specificity, the perception of organizational identity tends to differ significantly among individuals (Cloutier and Ravasi, 2020). Xu et al. based on role theory and social identity theory believe that CEO organizational identity enhances corporate charitable giving, while CFO organizational identity inhibits corporate charitable giving (Xu Y. et al., 2022). The organizational dimension is different from the individual dimension. Unlike the individual dimension, organizations, as representatives of group interests, tend to be oriented toward profit maximization, and in the organizational dimension, organizational identity has a tendency to be objective and interest-oriented. Organizational support theory considers organizational support and commitment to individual employees as a prerequisite for organizational commitment (Kurtessis et al., 2017). Organizations can use Internet technologies such as websites to communicate organizational identity to stakeholders, who are driven by certain factors such as reducing uncertainty to gain organizational legitimacy, maintaining self-consistency, or enhancing self-perception to choose to establish organizational identity with them (Brickson, 2005; Brown, 2017). New Organizational Identity Claims, a common method of changing organizational identity, need to both strengthen individual and organizational alignment and improve organizational Reputation plays an effective role (Mühlemann et al., 2022). In addition, much of the existing research has been based on employee and organizational identity claims. In addition, most existing research is based on employee and leadership perspectives, and organizational identity generally has a positive effect on innovation performance (Du and Wang, 2022). The higher the organizational identity, the higher the job satisfaction of the individual and the more inclined to defend the organizational interests (Mesmer-Magnus et al., 2018). Based on the digital empowerment context, this paper argues that the strong linkage of digital technology promotes the degree of individual effort to the organization also provides organizations with more opportunities and ways to reshape organizational identity, and due to the double-edged effect of digital empowerment, there are often deviations between the digital cognition of individuals and the digital cognition of the organization, and organizations reshape individuals’ cognition of digital through organizational identity.

Based on this, this paper proposes hypothesis 3: organizational identity plays a moderating role between organizational disordered climate and open innovation (H3).

### Organizational emotional capacity and open innovation

Emotion management has been one of the hot issues in the field of organizational behavior, but the existing studies mainly focus on the individual level and less on the organizational level (Arias-Perez et al., 2022). Emotional event theory is used to explain the relationship between the work environment and the behaviors expressed by emotions and attitudes (Weiss and Cropanzano, 1996). Emotional event theory is used to explain the relationship between the work environment and the behaviors expressed by emotion and attitude. Emotional event theory considers the work environment as a series of short-term, closely related discrete events that are a function of workplace characteristics (e.g., outcomes and origins) or are influenced by workplace characteristics that affect emotional responses and thus work behavior (Rantanen et al., 2011). These discrete events are characterized by organizational consistency, emotionality, and job relevance (Tu et al., 2020). Dorison et al. reviewed recent decades of research on emotions applied to organizational behavior and concluded that: (1) emotions are everywhere. It is unrealistic to expect employees to deal with emotions before they enter the workplace; (2) the influence of emotions on judgment and choice (JDM) is systematic and predictable; (3) the influence of emotions can be used or released to optimize decision making, and suppressing emotions is not a good solution strategy (McManus, 2021). At the organizational level, the diversity and variability of organizational emotions place higher demands on organizational emotional competence. Ashkanasy and Dorris constructed a five-dimensional model of organizational emotions: individual level, personnel interpersonal, interpersonal, inter-team and inter-organizational (Ashkanasy and Dorris, 2017). Organizational emotional competence is an organization’s ability to perceive, understand, monitor, adjust, and utilize organizational emotions to channel and reflect its emotions in organizational structures, practices, and processes (Huy, 1999, 2002; Huy et al., 2014). It includes six dimensions: identity dynamics, harmony dynamics, experience dynamics, play dynamics, expression dynamics, and inspiration dynamics. Huy believes that organizational emotional capability increases the likelihood of fundamental organizational change (Huy, 1999). There is a proliferation of research findings on the relationship between organizational emotions and innovation management, which have been explored by many scholars from different perspectives. People tend to be consistent and convergent in their emotions, and the transmission of emotions from person to person is like a viral infection (Hareli and Rafaeli, 2008; Zagenczyk et al., 2020). Barger and Grandy found that customers smiled more with the help of smiling customer service employees (Barger and Grandey, 2006). Based on emotional event theory and resource conservation theory, organizational emotions can perceive, integrate and apply individual emotions, dynamically adjust emotional strategies to regulate organizational emotional experiences, enhance organizational intellectual capital, and promote innovative activities (Vuori and Huy, 2022).

Based on this, this paper proposes hypothesis 4: Organizational emotional capabilities have a mediating role between digital empowerment and open innovation (H4).

First, according to the chain relationship of SOR theory, taking digital empowerment as the starting point, organizational Chaxu climate and organizational emotional ability as the organizational feedback when the organism faces the stimulus of digital empowerment, and open innovation as the response result, the following model is constructed. Second, The structural equation modeling method is used to study the mediation effect and regulation effect of the previous exploration. In addition, the necessity analysis method is used to analyze the necessity of all variables in the above model. Finally, according to the above research results, this paper gives the corresponding research conclusions and management inspiration.

**Figure 2:**
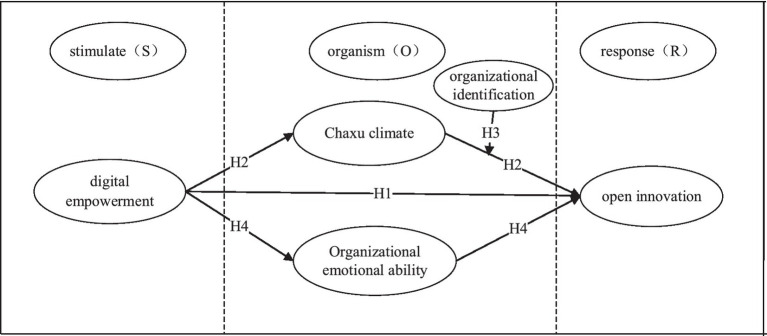
Open innovation performance impact mechanism model.

The structural equation model is a statistical method to analyze the relationship between variables based on the covariance matrix of variables, and is an important tool for multivariate data analysis. Selecting the structural equation model to study the impact of digital empowerment on the open innovation performance of enterprises from the perspective of SOR has the following advantages: first, structural equation analysis can consider and process multiple dependent variables at the same time; Second, structural equation models allow measurement errors for both independent and dependent variables. Variables can also be measured using multiple indicators. Third, the overall fit of different models to the same sample data can be calculated. In addition, the SOR chain model has a high degree of compatibility with the structural equation model, and scholars have achieved rich research results in many research fields using the structural equation model method and using the SOR theory as the framework. In summary, it is theoretically feasible to adopt the structural equation model and combine the SOR theoretical framework to explore the impact of digital empowerment on the open innovation performance of enterprises (Figure 2).

## Research design

The research object of this paper is high-tech enterprises. High-tech enterprises are often the main application objects of digital technology, considering the iterative nature of innovation, the main applicants for patent technology innovation are concentrated in high-tech enterprises. High-tech enterprises are ideal open innovation research samples. The sample enterprises are distributed in 21 provinces and cities such as Guangdong and Shanghai, which can comprehensively reflect the overall development of high-tech enterprises. The survey promises not to disclose corporate information, and follows the principle of anonymity of the questionnaire, which ensures the reliability of the content to a certain extent. This data collection is mainly based on alumni who have participated in courses related to digital transformation in the manufacturing industry as the contact person, and the questionnaire is distributed to their enterprises or relevant enterprises that meet the requirements. In order to avoid geographical restrictions, electronic questionnaires are distributed through channels such as Questionnaire Star, Credamo questionnaire platform and WeChat.

### Questionnaire design

In order to ensure the validity and reliability of the research results, this paper referred to the metrics that have been used and proved to be effective, and made appropriate screening and modification according to the actual situation, as shown in Table 1. In the questionnaire design, all items were measured by Likert scale. Respondents were scored on a scale of 1–7, with “1” indicating “strongly disagree” and “7” indicating “strongly agree.”

1. Digital empowerment, based on the research of Kerpen (2019) and Xu G. et al. (2022), this paper sets three questions to measure digital empowerment.
2. organizational identity, this paper refers to Ng (2015) and Mael and Ashforth (1992), which measured organizational identity with six question items.
3. Chaxu climate, this paper refers to Vveinhardt and Bendaraviciene (2022), three items were taken to measure the organizational Chaxu atmosphere by the reliability test.
4. Organizing emotional capacity, this paper refers to Huy (1999) and Hartmann et al. (2021) measured from 6 questions of identity dynamics, harmony dynamics, experience dynamics, play dynamics, expression dynamics, and inspiration dynamics.
5. Open innovation performance, referring to the research results of Lazzarotti et al. (2017), Han et al. (2020), and Hwang et al. (2021), measured from “new product development,” “R&D technology upgrading” and “patent application.”

**Table 1:** Reliability validity test.

Variables	Title item	Factor load	Cronbach’ a	CR	AVE
Digital empowerment	(FE0) has enhanced the management of all enterprise resources through the introduction of digital technology.	0.807	0.753	0.859	0.670
	(OE0) introduces digital technology in the business processes of production and sales or services of a company.	0.825			
	(SE0) makes the achievement of business goals more practical, feasible and tangible through digital technology.	0.822			
Organizational identity	(OD1) I think my values and the company’s are close.	0.635	0.748	0.844	0.581
	(OD2) I feel unhappy when outsiders criticize our company.	0.862			
	(OD3) I agree that the company and I are at the same loss, and the company’s failure is not beneficial to me.	0.873			
	(OD4) I agree that my success is my success and the company’s success is my success.	0.646			
Chaxu climate	(ODA0) In a team, some subordinates rise through the ranks much faster than others.	0.714	0.750	0.840	0.569
	(ODA1) In the team, the leader exchanges ideas more frequently with a portion of the regular employees.	0.831			
	(ODA2) In teams, the opinions of certain employees are influential in leadership decisions.	0.728			
	(ODA3) In teams, leaders tend to pass on information through regular employees.	0.738			
Organizing emotional capacity	(EXD0) companies where people can fully express their emotions without fear of being criticized or punished.	0.802	0.850	0.893	0.625
	(GD0) The company tolerates mistakes by first movers and shakers.	0.795			
	(HD0) Employees show certain reactions to others’ emotions.	0.735			
	(ID0) There are communication bridges between different group emotions.	0.836			
	(IDX0) Employees have a sense of identification with the organizational philosophy.	0.782			
Open innovation (OI)	(OI1) Companies are constantly developing new products.	0.791	0.753	0.857	0.667
	(OI2) An increase in the number of projects in which companies collaborate with external institutions for innovation.	0.829			
	(OI3) There has been a significant improvement in employees’ open and innovative thinking.	0.830			

### Research process and data collection

This study designed the questionnaire through the Credamo questionnaire platform and the questionnaire WJX. We distribute questionnaires through a total of three ways: on-site research, questionnaire star online platform and Credamo questionnaire platform. We require all participants to have work experience. In addition, we visited many high-tech enterprises in Shanghai, distributed questionnaires to grass-roots, middle and senior managers, and treated them anonymously. The question on the front page of the questionnaire is set as “whether you have work experience.” Only respondents who answer “Yes” can continue to answer the following questions. First, from 1 January 1 to 31 May 2022, 250 questionnaires were distributed and collected, and 38 invalid questionnaires were rejected after manual screening, and 212 valid questionnaires were finally obtained. The effective recovery rate of questionnaires was 84.8%. The distribution and characteristics of the survey respondents are shown in Table 2.

**Table 2:** Characteristics of survey respondents.

Features	Classification	Frequency	Frequency/%
Corporate history	Less than 5 years	9	4.25%
	6–10 years	59	27.83%
	11–15 years	64	30.19%
	16–20 years	52	24.53%
	More than 21 years	28	13.21%
Nature of ownership	State-owned	27	12.74%
	Private	157	74.06%
	Foreign investment	14	6.6%
	Joint venture	13	6.13%
	Other	1	0.47%
Enterprise size	1–99 people	51	24.06%
	100–499 people	97	45.75%
	500–1,000 people	39	18.4%
	More than 1,000 people	25	11.79%
Industry type	Consumer goods/home appliances	47	22.17%
	Automotive/machinery manufacturing	49	23.11%
	Electrical/optical	9	4.25%
	Chemical/biomedical	33	15.57%
	Semiconductor/electronic information	20	9.43%
	IT hardware/software	34	16.04%
	Other (please add)	20	9.43%
Position	Senior management	25	11.79%
	Middle management	50	23.58%
	Basic management	101	47.64%
	General staff	36	16.98%
Development stage	Early stage of business	5	2.36%
	Growth stage	93	43.87%
	Maturity stage	101	47.64%
	Transformation phase	13	6.13%
Academic qualifications	Specialty	23	10.85%
	Undergraduate	168	79.25%
	Graduate student and above	21	9.91%
	Other	0	0%
Personal growth environment	Rural	83	39.62%
	City	128	60.38%

## Data analysis results

### Reliability and validity analysis

This paper utilizes SPSS 26.0 and SmartPLS 3.0 for reliability and validity testing. Compared with other structural equation modeling software, SmartPLS3.0 is more advantageous in handling non-normal sample data and is more suitable for exploratory studies. The results of the reliability and validity tests are shown in Table 1. The Cronbach’s α coefficient of the internal consistency reliability index of each potential variable was greater than 0.7, and the CR values of the combined reliability were greater than 0.8, indicating that the measurement reliability of each potential variable of the scale met the requirements. Meanwhile, according to the standardized factor loading coefficient of each question item is greater than 0.6, and the average variance extraction (AVE) of all potential variables is greater than 0.5, which can indicate that all variables have good convergent validity.

According to the discriminant validity test in Table 3, it can be seen that the average variance extracted (AVE) of all potential variables is greater than the Pearson correlation coefficient between the variables, indicating that the discriminant validity of the measurement model has passed the test. The variance inflation factors (VIF) between variables are all less than 5, indicating that there is no serious problem of multicollinearity (VIF values ranged from 1.197 to 3.440). On the other hand, validated factor analysis (CFA) using SmartPLS 3.0 was performed in this paper, and the results showed a good model fit (SRMR of 0.120 and NFI of 0.642). Note: Bolded font is the AVE root value.

**Table 3:** Differential validity test.

Variables	AVE	Open innovation	Digital empowerment	Organizational identity	Chaxu climate	Organizing emotional capacity
Open innovation	0.667	**0.817**				
Digital empowerment	0.670	0.563	**0.818**			
Chaxu climate	0.569	0.167	0.417	**0.754**		
Organizing emotional capacity	0.625	0.600	0.623	0.382	**0.791**	
Organizational identity	0.581	0.457	0.450	0.355	0.643	**0.762**

The meaning of the bold values provided in table is AVE root value.

### Common method variance test

In order to avoid the problem of common method variation, this questionnaire was controlled ex ante as follows: (1) when creating the questionnaire, most of the questions were chosen with reference to the previous mature scales, and were expressed as clearly and concisely as possible; (2) when the questionnaire was distributed, the privacy protection of the respondents was ensured, and the voluntariness and anonymity of the respondents were also ensured; (3) when designing the questionnaire, some of the questions were disordered to enhance the authenticity of the data. On the other hand, according to Liang et al. (2007), this paper used SmartPLS3.0 to conduct the common method variance test. The mean substantive factor loadings were not greater than 1, so there were no items that needed to be removed. Since the amount of method variation is insignificant and small, in summary, the problem of common method variation of data in this paper is not serious.

### Path analysis

First, this paper uses the SmartPLS 3.0 structural equation model for empirical partial hypothesis testing. As can be seen from Table 4, the *R*^2^ value of the entire model is 0.460, indicating that the entire model explains 46.0% of the variance of open innovation performance, indicating that digital empowerment, Chaxu climate, and organizational emotional competence have strong explanatory power for open innovation. Table 4 shows the explained variance R^2^ values for the three dependent variables.

**Table 4:** R^2^ values for each dependent variable.

Endogenous variables	Open innovation	Chaxu climate	Organizing emotional capacity
R^2^	0.460	0.174	0.388
Adjusted R^2^	0.447	0.170	0.385

**Figure 3:**
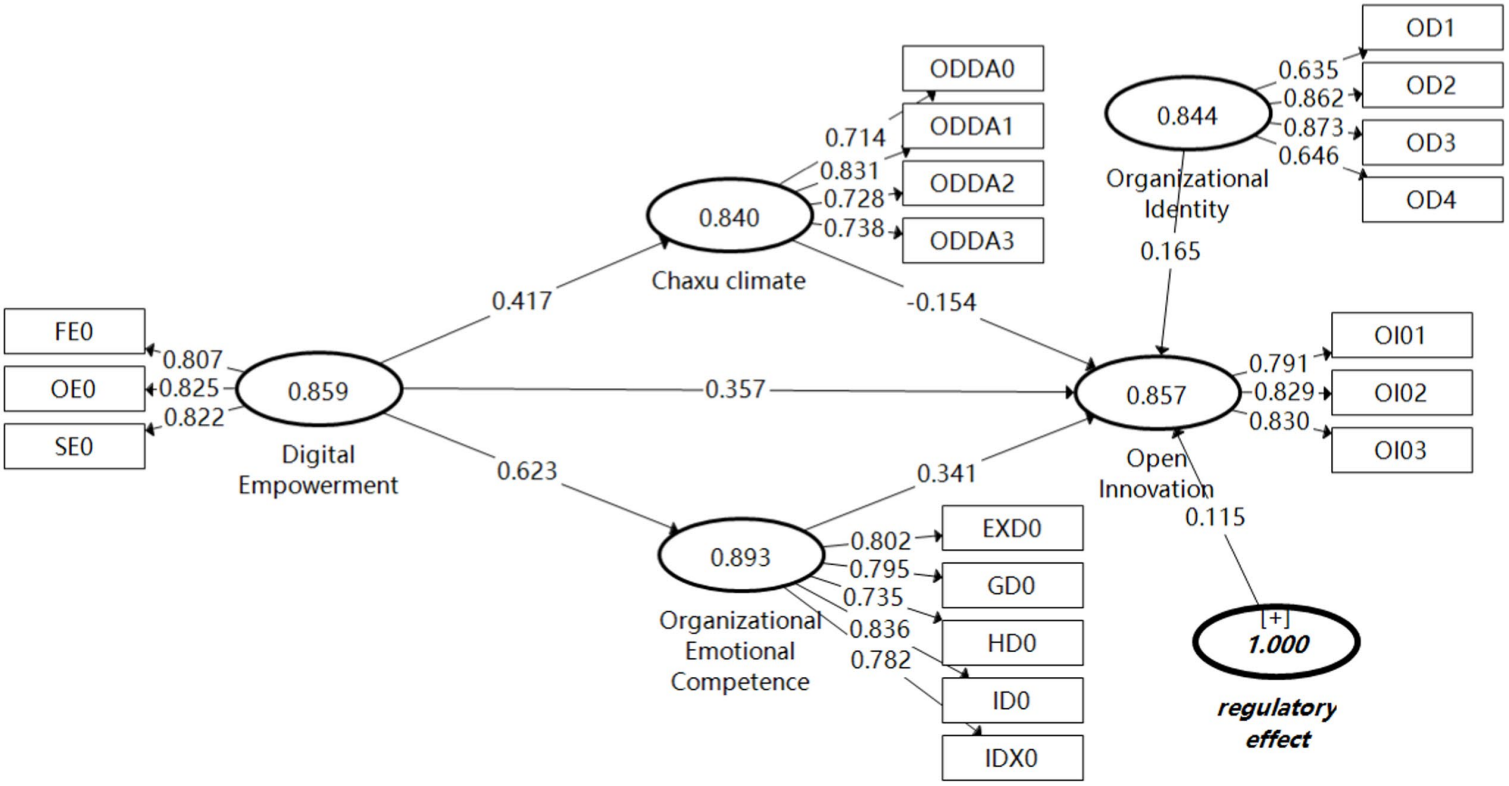
Path coefficient diagram.

It was found that (1) digital empowerment had a significant positive effect on open innovation (*β = 0*.357, *p* < 0.05). (2) Digital empowerment significantly and positively influenced organizational emotional competence and organizational Chaxu climate (β = 0.623, *p* < 0.05; β = 0.417, *p* < 0.05). (3) Organizational emotional competence had a significant positive effect on open innovation (β = 0.341, *p* < 0.05), and Chaxu climate had a significant negative effect on open innovation (β = −0.154, *p* < 0.05). In summary, the model supports the H1 hypothesis. Meanwhile, there may be a partial mediating effect of organizational emotional competence and Chaxu climate between digital empowerment and open innovation performance, and the mediating effect will be verified and analyzed below. Figure 3 shows the plot of path coefficients after running Bootstrapping program 5,000 times in SmartPLS 3.0 for sampling. Table 5 shows the hypothesis testing for the study in this paper.

**Table 5:** Research hypothesis testing.

Paths	Path factor	Sample Means	Standard deviation	T-statistic	95% confidence interval		*p*-value	Results
					Lower limit	Upper limit		
Digital empowerment → open innovation	0.357	0.361	0.080	4.494	0.203	0.514	0.000	Support
Digital empowerment → Chaxu climate	0.417	0.425	0.059	7.078	0.311	0.537	0.000	Support
Digital empowerment → organizational emotional competence	0.623	0.627	0.047	13.376	0.530	0.714	0.000	Support
Chaxu climate → OPEN innovation	−0.154	−0.152	0.062	2.469	−0.270	−0.024	0.014	Support
Organizational emotional competence → open innovation	0.341	0.333	0.091	3.742	0.151	0.502	0.000	Support
Organizational identity → open innovation	0.165	0.176	0.084	1.975	0.012	0.342	0.048	Support
Moderating effect1 → open innovation	0.115	0.118	0.047	2.451	0.025	0.210	0.014	Support

### Mediating effect test

In this paper, Bootstrapping is used to verify the mediating effect of organizational emotional competence and Chaxu climate on the relationship between open innovation and digital empowerment. Meanwhile, the number of repetitions of Bootstrapping is set to 5,000, and the results of the mediation effect analysis are shown in Table 6. from we can learn that the mediation effect is significant for all paths (the upper and lower limits of the confidence interval do not contain 0). Therefore, hypotheses H2 and H4 are supported, indicating that organizational emotional competence and organizational Chaxu climate play a mediating role between digital empowerment and open innovation. Specifically, Table 7 was used to determine the fullness of the mediating effect for all paths, distinguishing between a fully mediating effect or a partially mediating effect. According to Hair and Sarstedt (2019), we can judge the completeness of the mediating effect by VAF (Variance Accounted For), and the judgment criteria are VAF less than 20% indicates no mediating effect, VAF between 20 and 80% indicates partial mediating effect, and VAF greater than 80% indicates full mediating effect. In this paper, both the mediating effect and the direct effect are significant, so we further judge whether the mediating effect belongs to the complete mediating effect or the partial mediating effect, and by calculating the VAF, we get that the total VAF of the two mediating paths is 43.60% less than 80%, so the mediating effect in this paper belongs to the partial mediating effect. Similarly, the results of the mediation test for organizational differential climate and organizational emotional competence did not contain 0 at the 95% confidence interval (organizational differential climate: the lower limit was −0.124 and the upper limit was −0.010; organizational emotional competence: the lower limit was 0.092 and the upper limit was 0.324), therefore, both organizational differential climate and organizational emotional competence played a partial mediation effect.

**Table 6:** Significance tests for mediating effects.

Intermediary path	Path factor	Standard deviation	T-statistic	95 confidence interval		*P*-value	Results
				Lower limit	Upper limit		
Digital empowerment → Chaxu climate → open innovation	−0.064	0.029	2.241	−0.124	−0.010	0.025	Support
Digital empowerment → organizational emotional competence → open innovation	0.212	0.060	3.537	0.092	0.324	0.000	Support

**Table 7:** Mediated effects completeness test.

Intermediary path	Path factor	T-statistic	*P*-value	VAF	Total VAF	Intermediary effect
Digital empowerment → Chaxu climate → open innovation	−0.064	2.241	0.025	10.11%	43.60%	Partial agency effect
Digital empowerment → organizational emotional competence → open innovation	0.212	3.537	0.000	33.49%		
Digital empowerment → open innovation	0.357	4.494	0.000	56.40%	56.40%	Direct effect

### Moderating effect test

**Figure 4:**
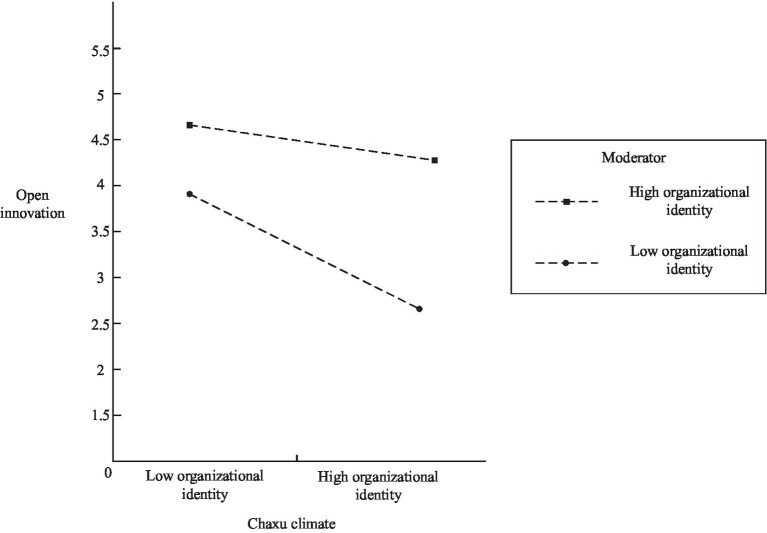
Moderating effect of organizational identity on organizational disorder climate and open innovation.

First, this paper used open innovation as the dependent variable, organizational identity as the moderating variable, and organizational climate as the independent variable, and used smartpls3.0 software to construct an interaction term moderating effect model between organizational identity and organizational climate, and the interaction term was significant. (β=0.115, *p*<0.05) indicating that there is a moderating effect between organizational climate and open innovation. h4 hypothesis is supported (Figure 4).

### Necessary condition analysis (NCA)

Regarding the necessity test of the antecedent variables, this paper uses R software loaded with NCA package to test the necessity of digital empowerment, organizational emotional competence, organizational Chaxu climate, and organizational identity on open innovation. The upper limit envelopes were obtained by two methods: upper limit regression (CR-FDH) method and upper limit envelope (CE-FDH) method. Tables 8, 9 show the results of the NCA analysis, in which both the effect size d and the value of p (*p* < 0.01) are referenced to determine to what extent the antecedent conditions are necessary for the results. The results of the NCA analysis show that digital empowerment and organizational emotional competence are necessary conditions for open innovation, and organizational Chaxu climate and organizational identity do not constitute necessary conditions for open innovation.

**Table 8:** NCA method single essential condition analysis results.

Conditional Variables	Methods	Accuracy/%	Upper limit area (ceiling zone)	Scope	Effect size	*P*-value
Digital empowerment	CR	98.1%	1.067	5.436	0.196	0.012
	CE	100%	1.260	5.436	0.232	0.002
Organizing emotional capacity	CR	97.2%	0.851	4.770	0.178	0.002
	CE	100%	0.955	4.770	0.200	0.000
Chaxu climate	CR	99.1%	0.397	5.824	0.068	0.506
	CE	100%	0.560	5.824	0.096	0.364
Organizational identity	CR	98.1%	0.864	5.436	0.159	0.034
	CE	100%	0.981	5.436	0.180	0.008

Effect size d, 0.0 ≤ d < 0.1: “low level”; 0.1 ≤ d < 0.3: “medium level.” *P*-values were determined using the permutation test in NCA analysis (number of resamples = 10,000).

**Table 9:** Results of bottleneck level analysis of NCA method.

Open Innovation	Digital empowerment	Organizational identity	Chaxu climate	Organizing emotional capacity
0	NN	NN	NN	NN
10	NN	NN	NN	NN
20	NN	NN	NN	NN
30	NN	NN	NN	NN
40	NN	NN	NN	NN
50	NN	NN	NN	NN
60	4.3	NN	NN	NN
70	26.6	10.0	NN	16.1
80	49.0	38.3	NN	44.0
90	71.3	66.6	28.6	71.8
100	93.6	94.9	95.6	99.7

Table 9 reports the bottleneck table of necessary conditions, from which it can be seen that achieving 80% open innovation requires 49% digital empowerment, 38.3% organizational buy-in and 44.0% organizational emotional competence, with an organizational Chaxu climate of NN unnecessary.

## Conclusions and recommendations

### Summary of finding

This paper empirically analyzes the impact mechanism of digital empowerment on open innovation from SOR perspective, explores the mediating role of organizational emotional competence and organizational differential climate as well as the moderating role of organizational identity, and the main conclusions of this paper are as follows:

1. Digital empowerment in the era of digital intelligence is an important means for organizations to carry out open innovation to gain competitive advantage (Yoo et al., 2012), but digital empowerment brings many benefits to organizations and also exacerbates the negative effect of Chaxu climate, which has a negative impact on open innovation. The development of digital technology has blurred organizational boundaries (Nambisan et al., 2017) and increased the frequency of communication and information exchange efficiency, which is conducive to the stability of the organization within the circle, thus exacerbating the disparity pattern. At the same time, due to network security issues and the concealment and security of information dissemination, people within circles tend to prefer traditional communication methods, which can intensify information exchange barriers between different circles, increase redundant information and waste of resources in the network, reduce the trust between organizations, and is not conducive to open innovation.
2. Based on the cognitive perspective, organizational differential climate mediates between digital empowerment and open innovation, and organizational identity moderates between organizational differential climate and open innovation, and the moderating effect is significant. The regulating effect of organizational identity can compensate for the negative impact of disordered organizational climate, and the dynamic and adjustable nature of the circle phenomenon and disordered pattern is thus verified (Vuori and Huy, 2022). The shape ability of organizational identity provides a feasible means for organizations to solve the problem of disorderly patterns (Du and Wang, 2022). The negative effects of digital empowerment are not caused by the limitations of digital technology itself, but rather digital empowerment amplifies the problems in organizational management to a certain extent, and is a contradiction between digital technology and traditional management models.
3. Organizational emotional capability plays a mediating role between digital empowerment and open innovation. Digital empowerment and organizational emotional capability as a necessary condition for open innovation and the negative effect of organizational disorderly climate caused by digital empowerment are not contradictory (Tu et al., 2020). Digital empowerment and organizational emotional capability are the necessary path and condition for organizations to achieve digital transformation, and digital empowerment can rapidly improve organizational operational efficiency and help organizations achieve open innovation.

### Management implications

1. With the advent of the digital intelligence era, enterprises should focus on the enhancement of digital empowerment thinking, remain sensitive to the opportunities of open innovation in the context of the digital intelligence era, and empower enterprise capabilities and open innovation with the help of digital intelligence technologies and intelligent platforms, so as to help enterprises gain competitive advantages (Wu et al., 2022). For enterprises, they should raise their digital empowerment cognition from the technical level to the strategic level, and develop a digital intelligence strategic policy that is in line with their own actual situation, so as to better adapt to environmental changes. In the era of digital intelligence, in order to enhance the depth and breadth of their own open innovation, enterprises should learn to make reasonable use of modern digital intelligence tools and digital intelligence platforms, make full use of the unique advantages brought about by technological change, choose a digital intelligence development strategy in combination with their own actual situation, and achieve twice the result with half the effort through digital empowerment of enterprise innovation performance.
2. The development of digital technology and the traditional management model have an adaptation bias, and the organization should pay attention to digital training and digital thinking education of the organization members while increasing the investment in digital construction. The organization should provide organizational support for members through various channels, enhance employees’ organizational commitment to shape a relational psychological contract, conduct regular digital education and organizational culture inculcation, reduce the disorderly atmosphere among teams, and enhance the team’s sense of cooperation, trust and overall awareness.
3. Enterprises should focus on the important role of organizational emotional capability and organizational identity in the process of digital empowerment. Organizational emotional capability and organizational identity are important bridges for digital empowerment to influence the improvement of open innovation. Facing the highly changing market environment, enterprises must enhance organizational emotional capability, improve organizational flexibility and organizational identity, identify and seize opportunities, and promote open innovation.

### Limitations and further research

This paper explores the influence mechanism of digital empowerment on open innovation based on previous studies, but there are other complex influencing factors that need to be investigated, and future studies can further explore the influence mechanism of digital empowerment on open innovation based on different perspectives. Finally, the research sample has certain geographical limitations, and future research can improve the geographical diversity and extensiveness of the sample sources to enhance the robustness and generalizability of the findings.

## Data availability statement

The original contributions presented in the study are included in the article/Supplementary material, further inquiries can be directed to the corresponding author.

## Author contributions

LY and LL: conceptualization and design and data acquisition and analysis. YL: data visualization, writing—original draft preparation, and writing—review and editing. All authors contributed to the article and approved the submitted version.

## Funding

This work was supported by the National Natural Science Foundation of China (NSFC), “Research on the value creation mechanism and transmission path of enterprise’s patent strategy” (71502110).

## Conflict of interest

The authors declare that the research was conducted in the absence of any commercial or financial relationships that could be construed as a potential conflict of interest.

## Publisher’s note

All claims expressed in this article are solely those of the authors and do not necessarily represent those of their affiliated organizations, or those of the publisher, the editors and the reviewers. Any product that may be evaluated in this article, or claim that may be made by its manufacturer, is not guaranteed or endorsed by the publisher.
